# Isobutylhydroxyamides from *Zanthoxylum bungeanum* and Their Suppression of NO Production

**DOI:** 10.3390/molecules21101416

**Published:** 2016-10-23

**Authors:** Yuan Wang, Chun-Huan Li, Bo Luo, Ya Nan Sun, Young Ho Kim, An-Zhi Wei, Jin-Ming Gao

**Affiliations:** 1 Shaanxi Key Laboratory of Natural Products & Chemical Biology, College of Science, Northwest A&F University, Yangling 712100, Shaanxi, China; angsoneaven@163.com (Y.W.); chunhuanli@nwsuaf.edu.cn (C.-H.L.); luobo2011@163.com (B.L.); 2 College of Forestry, Northwest A&F University, Yangling 712100, Shaanxi, China; 3 College of Pharmacy, Chungnam National University, Daejeon 305-764, Korea; yanansun@163.com (Y.N.S.); yhk@cnu.ac.kr (Y.H.K.)

**Keywords:** Huajiao, *Zanthoxylum bungeanum*, unsaturated alkylamides, isobutylhydroxyamides, sanshools, NO inhibitory activity, anti-inflammatory

## Abstract

Four new unsaturated aliphatic acid amides, named zanthoamides A–D (**1**–**4**), and eight known ones—tetrahydrobungeanool (**5**), ZP-amide A (**6**), ZP-amide B (**7**), ZP-amide C (**8**), ZP-amide D (**9**), ZP-amide E (**10**), bugeanumamide A (**11**), and (2*E*,7*E*,9*E*)-*N*-(2-hydroxy-2-methylpropyl)-6,11-dioxo-2,7,9-dodecatrienamide (**12**)—were isolated from the pericarps of *Zanthoxylum bungeanum*. The structures of these compounds were elucidated by extensive use of spectroscopic methods, including HRESIMS, 1D and 2D NMR analyses and comparison with previously reported data. Compound **4** contained a rare C_6_ fatty acid unit with an acetal group. Results revealed that compounds **1**, **5**, **6**, and **12** showed inhibitory effects on nitric oxide (NO) production in LPS-stimulated RAW 264.7 macrophages, with IC_50_ values of 48.7 ± 0.32, 27.1 ± 1.15, 49.8 ± 0.38, and 39.4 ± 0.63 µM, respectively, while the other compounds were inactive (IC_50_ > 60 μM). They could contribute to the anti-inflammatory effects of *Z. bungeanum* by suppression of NO production.

## 1. Introduction

The genus *Zanthoxylum* (family Rutaceae), commonly called “prickly ash”, comprises more than 200 species distributed worldwide. Phytochemical studies of this genus have revealed a variety of biologically active secondary metabolites, including alkaloids, aromatic and aliphatic amides, lignans and coumarins with antitumor, anti-inflammation, and anesthetic properties [1,2,3]. *Zanthoxylum bungeanum* Maxim is an aromatic tree and shrub, which is native to the provinces of Sichuan, Shaanxi, Yunnan, Guizhou, Guanxi, and Guandong in southwestern China. The fruits of this species, called “da hong pao” (big red robe), are the most popular red huajiao commercial product. Red huajiao, the pericarps of the fruits of *Z. bungeanum* have been utilized as a pungent foodstuff and also as a kind of traditional Chinese medicine for the treatment of vomiting, toothache, stomachache, abdominal pain, and diarrhea [4]. Previous phytochemical research on *Z. bungeanum* has focused on the essential oils, coumarins, flavonoids, aliphatic amides (classified as sanshools), and alkaloids of the fruits and leaves [5,6,7,8,9].The huajiao extracts have great potential for applications in savory and sweet goods and beverages. Some of the aliphatic acid amides display antioxidant activities, modulatory effects on relaxing the circle muscle of the gastric body (β- and γ-sanshool), anthelmintic, cytotoxic properties [10,11,12], as well as anti-type-1 diabetes (i.e., γ-sanshool) [13] and anti-tumor effects [14]. In addition, three aliphatic acid amides (β-, γ- and hydroxy-β-sanshool) exhibited human acyl-CoA: cholesterol acyltransferase inhibitory activities [15], and tumuramide C, ZP-amide A, and ZP-amide D exerted significant effects on PPAR transactivational activity [16]. Most recently, Hofmann et al. [1] have shown that the all-*trans*-configured amides hydroxy-β-sanshool and hydroxy-γ-isosanshool from *Z. piperitum* induced a numbing and anesthetic sensation. Our recent studies have showed that several alkylamides from cultivated *Z. bungeanum* pericarps possessed nerve growth factor-potentiating activity [17].

**Figure 1:**
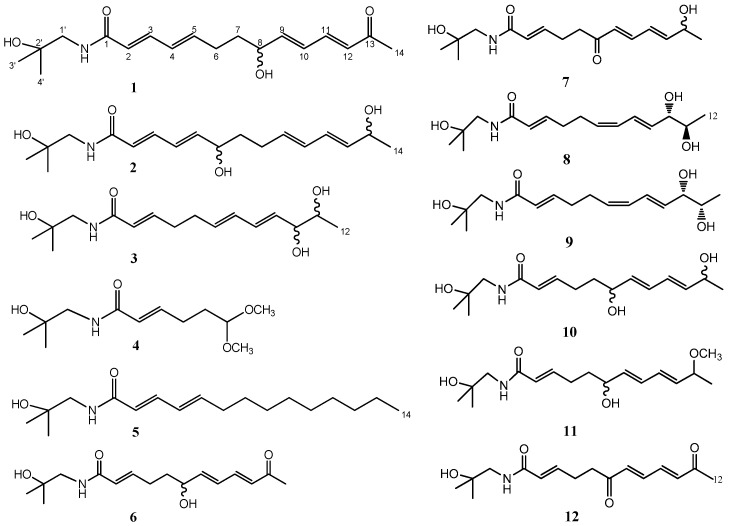
Chemical structures of compounds **1**–**12**.

As part of our further search for bioactive substances from medicinal plants [18,19,20,21], our bioassays indicated that the CHCl_3_-soluble material of a crude EtOH extract of *Z. bungeanum* pericarps displayed inhibitory activity against nitric oxide (NO) production in LPS-activated RAW264.7 macrophages. Further phytochemical investigation of the CHCl_3_-soluble material of *Z. bungeanum* led to the isolation of four new alkylamides, named zanthoamides A–D (**1**–**4**), and eight known ones **5**–**12** (Figure 1). Here, we describe the isolation and structure elucidation of the compounds **1**–**12** and their NO inhibitory activity in LPS-stimulated RAW 264.7 cells.

## 2. Results and Discussion

### 2.1. Compound Characterization

The CHCl_3_-soluble extract of the 95% ethanolic residue of the air-dried *Z. bungeanum* fruits was subjected to various separation procedures, leading to the isolation of four new isobutylhydroxyamides **1**–**4**, together with eight known congeners including tetrahydrobungeanool (**5**) [5], ZP-amide A (**6**) [7], ZP-amide B (**7**), ZP-amide C (**8**), ZP-amide D (**9**), ZP-amide E (**10**) [7], bugeanumamide A (**11**) [16], and (2*E*,7*E*,9*E*)-*N*-(2-hydroxy-2-methylpropyl)-6,11-dioxo-2,7,9-dodecatrienamide (**12**) [4] (Figure 1). The structures of known compounds **5**–**12** were identified by their spectroscopic data and comparison with literature data. All isolated compounds possessed maximal UV absorptions near 230 nm, consistent with the presence of a conjugated amide [14].

Compound **1** was obtained as a pale yellow syrup. Its molecular formula was determined as C_18_H_27_NO_4_ by HRESIMS at *m*/*z* 344.1810 [M + Na]^+^ (calcd. for C_18_H_27_NO_4_Na, 344.1838), indicating six degrees of unsaturation. The IR spectrum displayed characteristic absorptions of hydroxyl and amide NH (3370 cm^−1^) and amide (1665 cm^−1^) groups. The ^13^C-NMR and DEPT spectra (Table 1) demonstrated 18 carbon resonance, which were classified as three methyls (δ_C_ 27.2 × 2, 27.0), three methylenes with one of them occurring relatively downfield (δ_C_ 51.2), nine methines, including one oxygenated carbon (δ_C_ 71.8), and eight olefinic ones (δ_C_ 148.2, 145.4, 143.2, 142.4, 131.2, 130.3, 128.9, 123.2), an amide carbon (δ_C_ 169.4) and a ketonic carbon (δ_C_ 201.5), and one oxygenated quaternary carbon (δ_C_ 71.7). These data were consistent with the resonances observed in the ^1^H-NMR spectrum. The ^1^H-NMR spectrum of **1** revealed three tertiary methyl signals at δ_H_ 1.18, 1.18, and 2.28 (each 3H of singlet). Meanwhile, eight olefinic protons at δ_H_ 6.01, 7.14, 6.18, 6.14, 6.27, 6.44, 7.29, and 6.15, and an oxygenated methine proton at δ_H_ 4.22 (m), were clearly visible. Other overlapping proton resonances occurred between δ_H_ 1.66 and 2.28, resulting from either methyl or methylene protons. Comparison of the ^1^H- and ^13^C-NMR data (Table 1) of **1** with two known compounds (**5** and **6**) indicated that **1** was an unsaturated fatty acid amide bearing an *N*-hydroxylisobutyl moiety.

**Figure 2:**
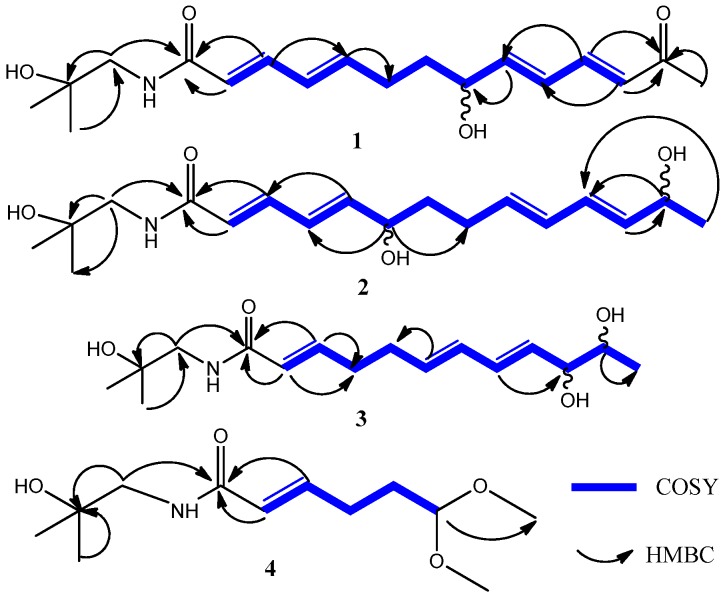
Selected 2D NMR correlations for compounds **1**–**4**.

Detailed analysis of the ^1^H and ^13^C-NMR data, along with the COSY, HSQC, and HMBC spectra (Figure 2) led to the conclusion that **1** was structurally similar to ZP-amide A (**6**).

The major difference was one more olefinic bond between the C-3 and C-6 positions in the aliphatic chain of **1** than in ZP-amide A (**6**), which was consistent with its molecular formula and degree of unsaturation. The ^1^H-^1^H COSY spectrum (Figure 2) indicated vicinal correlations of H-2/H-3/H-4/H-5/H-6/H-7/H-8/H-9/H-10/H-11/H-12 and the HMBC experiment showed correlations from H-1′, H-2, and H-3 to C-1, H-9 to C-8 (δ_C_ 71.8), and H-11, H-12 and H_3_-14 to C-13 (Figure 2), thus suggesting that the locations of the amide, hydroxyl, ketonic carbons to be at C-1, C-8 and C-13, respectively, and the connectivity between the aliphatic acid and the amine moieties. Additionally, the geometry of the C_4_/C_5_ olefinic bond was deduced to be *trans*-configured, like those of C_2_/C_3_, C_9_/C_10_, and C_11_/C_12_ olefinic bond, from the coupling constant (*J*_H-4/H-5_ = 15.7 Hz). The optical inactivity of this compound indicated that **1** was a racemic mixture. Consequently, compound **1** was identified as (2*E*,4*E*,9*E*,11*E*)-*N*-(2-hydroxy-2-methypropyl)-8-hydroxy-13-oxo-2,4,9,11-tetradecatetraenamide, and named zanthoamide A.

Compound **2** had a molecular formula C_18_H_29_NO_4_ by HRESIMS at *m*/*z* 346.1961 [M + Na]^+^, indicating five degrees of unsaturation. Comparison of the 1D NMR (Table 1) spectroscopic data of **2** with those of **1** clearly disclosed that **2** was also an aliphatic acid amide. The obvious difference between the two compounds was that the ketone carbonyl at C-13 (δ_C_ 201.5) in **1** was replaced by a hydroxyl group in **2**, which was confirmed by the upfield shift of C-13 (δ_C_ 68.8) in **2**, in combination of the ^1^H-^1^H COSY correlations of H_3_-14/H-13/H-12 and the HMBC correlations from H-13 to C-11 (δ_C_ 145.4), and H-12 (δ_H_ 6.18) to C-13 in **2**. Moreover, the HSQC spectrum suggested the hydroxyl group to be ascribable to C-6 in **2** rather than to C-8 in **1**, as evident from the large downfield chemical shift of C-6 (δ_C_ 72.4) and upfield shift of C-8 (δ_C_ 29.9) observed in the ^13^C-NMR spectrum of **2**. This was further confirmed by the COSY correlations of H-2/H-3/H-4/H-5/H-6. The assignments of all proton and carbon signals were fully made by 2D (^1^H-^1^H COSY, HSQC, and HMBC) NMR data. The optical inactivity of this compound indicated that **1** was a racemic mixture. Thus, **2** was identified as (2*E*,4*E*,9*E*,11*E*)-*N*-(hydroxy-2-methypropyl)-8,13-dihydroxy-2,4,9,11-tetradeca-tetraenamide, and named zanthoamide B.

Compound **3** was obtained as a colorless syrup. Its molecular formula C_16_H_27_NO_4_ was determined by HRESIMS at *m*/*z* 320.1813 [M + Na]^+^ (calcd. for C_16_H_27_NO_4_Na, 320.1838). The ^1^H and ^13^C-NMR data of compound **3** were very similar to those of ZP-amide C (**8**). The only differences between the two compounds were that the *cis*-configured olefin at C-6 and C-7 at δ_H_ 5.40 (*dt*, *J* = 11, 7 Hz, H-6) in **8** was replaced by a *trans*-configured one at δ_H_ 5.70 (*dt*, *J* = 15.3, 7.0 Hz, H-6) in **3**. The structure of this compound was confirmed by detailed analysis of the 2D NMR data including its HSQC, HMBC, and ^1^H–^1^H COSY spectra. The optical inactivity of this compound indicated that it was a racemic mixture. The relative configuration of the two asymmetric carbons still remains to be determined. Consequently, **3** was established to be (10*,11*)-(2*E*,6*E*,8*E*)-10,11-dihydroxy-*N*-(2-hydroxy-2,6,8-dodecatrienamide, and named zanthoamide C.

Compound **4** was also obtained as a colorless syrup having a molecular formula C_12_H_23_NO_4_, as deduced from HRESIMS at *m*/*z* 268.1505 [M + Na]^+^ (calcd. for C_12_H_23_NO_4_Na, 268.1525). The ^1^H and ^13^C-NMR spectra of **4** (Table 2), compared with those of **3**, clearly showed that **4** was also an aliphatic acid amide, which contained a rare C_6_ fatty acid with an acetal group, as evident from the ^1^H–^1^H COSY correlations of H-2 (δ_H_ 6.02)/H-3(δ_H_ 6.80)/H_2_-4(δ_H_ 2.25)/H_2_-5(δ_H_ 1.74)/H-6(δ_H_ 4.39) as well as from the ^1^H and ^13^C-NMR data of an acetal group (δ_H_ 4.39, *J* = 5.7 Hz, H-6; δ_C_ 105.5, C-6; δ_H_ 3.33 and δ_C_ 53.6, 2 × OCH_3_). The presence of the acetal moiety at C-6 was supported by the HMBC correlation of OCH_3_ (δ_C_ 53.6) with H-6 (δ_H_ 4.39) obtained in MeOH-*d*_4_ solvent. However, due to overlapping between the methoxyl and MeOH-*d*_4_ signals, consequently, we measured the ^1^H-NMR spectrum (see Figure S25 in Supplementary Materials) with DMSO-*d*_6_ as solvent, in which the six protons of the two methoxyl groups as a strong singlet were observed at δ_H_ 3.22. Therefore, compound **4** was identified as (2*E*)-6,6-dimethoxy-*N*-(2-hydroxy-2-methylpropyl)-2-hexenamide, and named zanthoamide D.

Unsaturated fatty acid amides are characteristic constituents of the genus *Zanthoxylum*, which contains more than 50 such compounds [16,22,23,24,25]. Nearly all aliphatic acid amides from the *Zanthoxylum* species have been isolated as racemates, and their absolute configurations have yet to be determined [16]. To the best of our knowledge, compound **4** is the first example of a fatty acid amide containing a C_6_ fatty acid unit found in the genus *Zanthoxylum*. It should be noted that **4** possesses an acetal group in its structure. Owing to contact with methanol during the extraction and purification processes, it may be a new fatty acid amide artifact presumably formed during these processes.

### 2.2. Biological Activity Assays

Compounds **1**–**12** were evaluated for their inhibitory activities against nitric oxide (NO) production in LPS-activated RAW264.7 cells and their cytotoxicities against two human cancer cell lines [26,27]. This assay indicated that compounds **1**, **5**, **6**, and **12** exhibited inhibitory activities against NO production in LPS-activated RAW264.7 macrophages, with IC_50_ values of 48.7 ± 0.32, 27.1 ± 1.15, 49.8 ± 0.38, and 39.4 ± 0.63 μM, respectively, while the other compounds were inactive (IC_50_ > 60 μM) (Table 3). Among them, **5** exhibited the highest inhibitory activity. The observations suggested that the unsaturated longer aliphatic chain without oxygen functions appears to play an important role in inhibiting NO production (**5** vs. **1** and **2**).

Additionally, the cytotoxicity of each compound was examined. Cell viability was measured by the MTT colorimetric assay. Compounds **1**–**12** had no significant cytotoxicity in LPS-stimulated RAW 264.7 cells at concentrations up to 50.0 μM (data not shown). At 50 μM, the cytotoxic activities against human colon cancer (HCT116) and human prostate cancer (PC-3) cells were also tested, and none of them exhibited detectable cytotoxicity.

It has been reported that four amides—ZP-amide A (**6**), ZP-amide C (**7**), ZP-amide D (**9**), and ZP-amide E (**10**)—inhibited the growth of a neurofibromatosis type 1 (*NF1*)-and p53-deficient mouse glioma cell line at non-cytotoxic concentrations [14]. In addition, ZP-amide A (**6**) and ZP-amide D (**9**) exerted significant anti-inflammatory effects through enhancing the peroxisome proliferator-activated receptor (PPAR) transactivational activity with EC_50_ values of 19.1 and 12.0 μM, respectively [16]. The findings further suggest that alkylamides are the major anti-inflammatory components of the edible spice, *Z. bungeanum*, which was also substantiated by our observations that some compounds (i.e., **5** and **12**) display NO-inhibitory activity. It is well known that NO is a crucial cellular-signaling molecule associated with several physiological and pathological processes [28]. It is therefore a fundamental component in the fields of neuroscience, physiology, and immunology [29]. NO has been shown to activate NF-κB in peripheral blood mononuclear cells, an important transcription factor in iNOS gene expression in response to inflammation [30]. So suppression of NO production was a direct indicator of those compounds to resist inflammation.

## 3. Experimental Section

### 3.1. General Procedures

Optical rotations were measured using an Autopol III automatic polarimeter (Rudolph Research Analytical, Hackettstown, NJ, USA). UV spectrum were obtained on an Evolution-300 UV-visible spectrophotometer (Thermo Fisher Scientific Inc., Waltham, MA, USA) and a Tensor 27 FT-IR spectrometer (Bruker Optics, Germany) in KBr pellets, respectively. NMR spectra were measured on an Avance III 500 instruments (Bruker Daltonics Inc., Bremen, Germany), with tetramethylsilane (TMS) as an internal standard at room temperature. ESI-MS were performed on a LTQ Fleet instrument (Thermo Fisher Scientific Inc., Waltham, MA, USA), and HRESIMS were obtained on a Thermo Fisher Scientific Q-TOF mass spectrometer (Thermo Fisher Scientific Inc., Waltham, MA, USA). Semipreparative HPLC was performed on a Waters 1100 liquid chromatography system (Waters Corp., Milford, MA, USA) equipped with a Hypersil BDS C_18_ column (4.6 mm × 250 mm and 10.0 mm × 250 mm, 5 μm, (Thermo Fisher Scientific Inc., Waltham, MA, USA). Column chromatography (CC) was performed on silica gel (90–150 μm) (Qingdao Marine Chemical Inc., Qingdao, China), Sephadex LH-20 (40–70 μm; Amersham Pharmacia Biotech AB, Uppsala, Sweden), and Lichroprep RP-18 gel (40–63 μm; Merck, Darmstadt, Germany). GF_254_ plates (Qingdao Marine Chemical Inc.) were used for thin-layer chromatography (TLC). Preparative TLC (PTLC) was carried out on silica gel 60 GF_254_ (Qingdao Marine Chemical, Ltd.). Finally, compounds were visualized under UV light (254 nm) and by dipping into 10% H_2_SO_4_ in ethanol followed by heating.

### 3.2. Plant Materials

The pericarps of *Z. bungeanum* were collected from Feng County (Shaanxi, China), in July 2013 and authenticated by Prof. Zai-Min Jiang (Plant Laboratory, College of Life Sciences, Northwest A&F University, Shaanxi, China). A voucher specimen (LXY-0156) was deposited in our laboratory.

### 3.3. Extraction, Isolation and Purification

The air-dried and powdered pericarps of *Z. bungeanum* (1.0 kg) were extracted three times with 95% ethanol (2.5 L × 3) for 24 h. A crude extract was partitioned between MeOH and petroleum ether (1:1) to afford the MeOH-soluble extract (73.0 g), which was dissolved in water. The aqueous syrup was adjusted to pH 2 with 2% HCl and then filtered. The filtrate, adjusted to pH 9 with 20% NaOH, was extracted with CHCl_3_ to afford an extract (39.2 g). The extract was subjected to silica gel column chromatography (CC) eluting with petroleum ether (PE)-EtOAc (5:1, 2:1, 1:1 and 1:2) to give two fractions (A and B). Fraction A (28.6 g) was separated by silica gel column with CHCl_3_-MeOH (50:1, 40:1, 30:1) to afford three fractions (Fr.A1-A3). Chromatography of Fr.A1 (2.88 g) on silica gel column with PE-EtOAc (2:1, 1:1, 1:2, 1:3, 1:4) gave four fractions (Fr.1-4). Fr1. (622 mg) was separated by CC on Sephadex LH-20 (MeOH) to obtain three fractions (Fr.1-1 to Fr.1-3). Fr.1-1 was purified by PTLC (CHCl_3_-MeOH 20:1) and HPLC on C_18_ column (4.6 × 250 mm) (60% MeOH) to yield **4** (5.2 mg, t*_R_* 7.8 min). Similarly, chromatographies of Fr.1-2 on silica gel column (CHCl_3_-MeOH 35:1), LH-20 (MeOH) and HPLC (55% MeOH) gave **6** (15.9 mg), **8** (18 mg), and **9** (20 mg). Fr.2 (3.70 g) was subjected to RP-C18 with MeOH-H_2_O (10:90-100:0) to provide two fractions (Fr.2-1 to Fr.2-2). Fr.2-1 (1.92 g) was successively separated by CC on RP-C18 (40% MeOH), PTLC (CHCl_3_-MeOH 25:1) and HPLC (60% MeOH) to give **12** (120.9 mg) and **7** (26 mg). Meanwhile, Fr.2-2 (600 mg) was separated by CC on LH-20 (MeOH), silica gel (CHCl_3_-MeOH 20:1) and then HPLC (55% MeOH, flow rate 10 mL/min) to yield **11** (5.1 mg). Fr.3 (6.32 g) provided two subfractions through CC on RP-C18 (MeOH), whose first subfraction was successively subjected to LH-20 (MeOH), RP-C18 (40% MeOH), silica gel CC (CHCl_3_-MeOH 30:1) and HPLC (65% MeOH) to afford **1** (5 mg), **2** (20 mg), and **3** (10 mg). The other subfraction was separated by HPLC (60% MeOH) to obtain **10** (16 mg). Compound **5** (20 mg) was obtained from Fr.4 (19.6 g) through LH-20 (MeOH), silica gel (petroleum ether-EtOAc 2:1), and RP-C18 (80% MeOH) CC.

### 3.4. Identification

*Zanthoamide A* (**1**). Pale yellow oil; ${\left[\alpha \right]}_{D}^{30}$ ±0 (*c* 0.10, MeOH); UV (MeOH) λ_max_nm (logɛ): 268 (1.313); IR (film) ν_max_ 3370, 2972, 2934, 1665, 1545,1382, 1268, 1178, 1028, 980, 907, 768, 586 cm^−1^; ^1^H- and ^13^C-NMR data, see Table 1; positive-ion HRESIMS *m*/*z* 344.1810 [M + Na]^+^ (calcd for C_18_H_27_NO_4_Na, 344.1838).

*Zanthoamide B* (**2**). Pale yellow syrup; ${\left[\alpha \right]}_{D}^{30}$ ±0 (*c* 0.12, MeOH); UV (MeOH) λ_max_nm (logɛ): 231 (1.342); IR (film) ν_max_ 3377, 2973, 2932, 1663, 1549,1382, 1267, 1178, 1028, 907, 768, 624 cm^−1^; ^1^H- and ^13^C-NMR data, see Table 1; positive-ion HRESIMS *m*/*z* 346.1961 [M + Na]^+^ (calcd for C_18_H_29_NO_4_Na, 346.1994).

*Zanthoamide C* (**3**). Colorless syrup, ${\left[\alpha \right]}_{D}^{29}$ ±0 (*c* 0.12, MeOH); UV (MeOH) λ_max_nm (logɛ): 230 (1.342); IR (film) ν_max_ 3354, 2973, 2930, 1710, 1667, 1634, 1549, 1380, 1221, 1178, 1074, 1030, 980, 907, 768, 651 cm^−1^; ^1^H- and ^13^C-NMR data, see Table 1; positive-ion HRESIMS *m*/*z* 320.1813 [M + Na]^+^ (calcd for C_16_H_27_NO_4_Na, 320.1838).

*Zanthoamide D* (**4**). Colorless syrup ${\left[\alpha \right]}_{D}^{30}$ ±0 (*c* 0.16, MeOH); UV (MeOH) λ_max_nm (logɛ): 268 (1.313); IR (film) ν_max_ 3377, 2973, 2932, 1669, 1547,1463, 1267, 980, 615 cm^−1^; ^1^H and ^13^C-NMR data, see Table 2; positive-ion HRESIMS *m*/*z* 268.1505 [M + Na]^+^ (calcd for C_12_H_23_NO_4_Na, 268.1525).

### 3.5. Nitric Oxide Production Inhibition Assay

RAW 264.7 cells were seeded at a density of 5 × 10^5^-cells/well in 24 well plates and incubated for 12 h at 37 °C and 5% CO_2_ [26]. Then media of each well were aspirated and fresh FBS-free DMEM media were replaced. Compounds **1**–**12** (20 µM) were prepared in FBS-free DMEM to give a total volume of 500 µL in each well of a microtiter plate. After 1 h treatment, cells were stimulated with 100 ng/mL of LPS for 24 h. Nitrite concentrations were determined from a standard curve using sodium nitrite at concentrations ranging from 0 to 120 µM. The absorbance was measured at 540 nm in a microplatereader (Biotek, Winooski, VT, USA). The amount of nitrite inthe media was calculated from sodium nitritestandard curve. The data show the mean ± S.D. of three independent experiments. *p* < 0.05, and *p* < 0.001. The values expressed are means of three replicate determinations ± standard deviation. The data were evaluated with SPSS 20.0 (SPSS Inc., Chicago, IL, USA).

### 3.6. Cytotoxicity Assays

The cytotoxicity assay was performed in 96-well microplates using the 3-(4,5-dimethylthiazol-2-yl)-2,5-diphenyltetrazolium bromide (MTT) method [27]. Compounds **1**–**12** were evaluated for their cytotoxicity against human colon cancer (HCT116) and human prostate cancer (PC-3) cells. Cells were cultured in RPMI-1640 or in DMEM medium (Hyclone, Kerrville, TX, USA) supplemented with 10% fetal bovine serum (Hyclone) in 5% CO_2_ at 37 °C.

## 4. Conclusions

In conclusion, we have identified 12 lipophilic alkyamides of the sandshool class from the pericarps of *Z. bungeanum*, including four new ones **1**–**4**, which we have named zanthoamides A-D. NO inhibitory activity was observed for these alkyamides (compounds **1**, **5**, **6**, and **12**) in LPS-stimulated RAW 264.7 cells, of which **5** was the best inhibitor. The results demonstrated that these fatty acid amides might play important roles in anti-inflammatory activity of the pericarps of *Z. bungeanum*.

## Figures and Tables

**Table 1:** ^1^H-NMR (500 MHz) and ^13^C-NMR (125 MHz) data for **1**–**3** in MeOH-*d*_4_ (δ ppm).

No.	1		2		3	
	δ_H_ (*J* in Hz)	δ_C_^a^	δ_H_ (*J* in Hz)	δ_C_^a^	δ_H_ (*J* in Hz)	δ_C_^a^
1		169.4		169.5		169.0
2	6.01 d (15.1)	123.2	6.0 m	123.1	5.29 d (15.0)	125.1
3	7.14 dd (15.7, 10.7)	142.4	7.14 dd (15.1, 10.7)	142.4	6.80 dt (15.6, 6.5)	145.0
4	6.23 dd (15.7, 10.7)	130.3	6.25 dd (15.1, 10.7)	130.1	2.30 dt (12.5, 6.5)	32.8
5	6.18 dt (15.7, 10.2)	143.2	5.70 dd (15.1, 7.4)	143.1	2.25 dt (12.5, 6.5)	32.4
6	2.28 overlap	29.8	6.09 dd (13.1, 7.4)	72.4	5.70 dt (15.3, 7.0)	133.2
7	1.66 m	37.0	1.63 m	37.4	6.12 dd (15.3, 10.6)	132.1
8	4.22 q (6.0)	71.8	2.23 td (14.8, 7.4)	29.9	6.23 dd (15.3, 10.6)	131.9
9	6.28 dd (15.6, 11.4)	148.2	6.15 dt (15.8, 7.4)	137.0	5.68 ddd (22.0, 14.6, 7.0)	134.0
10	6.44 dd (15.6, 10.8)	128.9	6.22 d (14.3)	131.1	3.90 m	77.5
11	7.29 dd (15.6, 10.8)	145.4	6.22 d (14.3)	138.6	3.66 m	71.6
12	6.15 d (15.6)	131.2	5.75 m	129.8	1.13 d (6.4)	18.6
13		201.5	4.27 q (6.4)	68.8		
14	2.28 overlap	27.0	1.23 d (6.4)	23.6		
1′	3.26 s	51.2	3.26 s	51.1	3.25 s	51.1
2′		71.7		71.6		71.7
3′/4′	1.18 s	27.2	1.18 s	27.2	1.17 s	27.2

^a^ The assignments are based on HSQC, HMBC, and ^1^H-^1^H COSY experiments.

**Table 2:** ^1^H- (500 MHz) and ^13^C- (125 MHz) NMR Data of Compound **4** (δ ppm).

No.	δ_H_ Multi. (*J* in Hz) ^a^	δ_C_^a^	δ_H_ Multi. (*J* in Hz) ^b^
1		169.0	
2	6.02 dt (15.4, 1.5)	124.9	6.03 d (15.5)
3	6.80 dt (15.4, 6.9)	145.1	6.61 m
4	2.25 td (8.4, 1.5)	28.1	2.13 dd (14.9, 6.6)
5	1.74 m	32.4	1.63 dd (14.4, 6.5)
6	4.39 t (5.7)	105.5	4.34 t (5.5)
1′	3.25 s	51.1	3.07 d (6.0)
2′		71.6	
3′/4′	1.17 s	27.2	1.04 s
2 × OCH_3_	3.33 ^c^	53.6	3.22 s

^a^ MeOH-*d*_6_ was used solvent; ^b^ DMSO-*d*_6_ was used solvent; ^c^ This signal was overlapped with solvent peaks.

**Table 3:** Inhibitory effects of compounds **1**–**12** on NO production by LPS-stimulated RAW264.7 cells.

No.	IC_50_ Values (µM)
**1**	48.7 ± 0.32
**2**	NT
**3**	NT
**4**	NT
**5**	27.1 ± 1.15
**6**	49.8 ± 0.38
**7**	NT
**8**	112.9 ± 0.91
**9**	62.3 ± 1.12
**10**	NT
**11**	NT
**12**	39.4 ± 0.63
Dexamethasone	1.54 ± 0.07

Dexsamethasone was used as positive control. Data presented is the mean ± S.D. of samples run in triplicate. Compared with control *p* < 0.001. NT: not tested.

## Supplementary Materials

NMR and HRESIMS spectra for compounds **1**–**4** can be accessed at: http://www.mdpi.com/1420-3049/21/10/1416/s1.
