# Supplementary material for: Isobutylhydroxyamides from Zanthoxylum bungeanum and Their Suppression of NO Production
Source: Molecules. 2016 Oct 23;21(10):1416. doi: 10.3390/molecules21101416 (PMC6274162; doi:10.3390/molecules21101416)
Supplement: Supplementary file 1 [file molecules-21-01416-s001.pdf]

Molecules **2016**, *21*, 1416; doi:10.3390/molecules21101416

Supplementary Materials: Isobutylhydroxyamides from *Zanthoxylum bungeanum* and Their Suppression of NO Production

Yuan Wang, Chun-Huan Li, Bo Luo, Ya Nan Sun, Young Ho Kim, An-Zhi Wei and Jin-Ming Gao

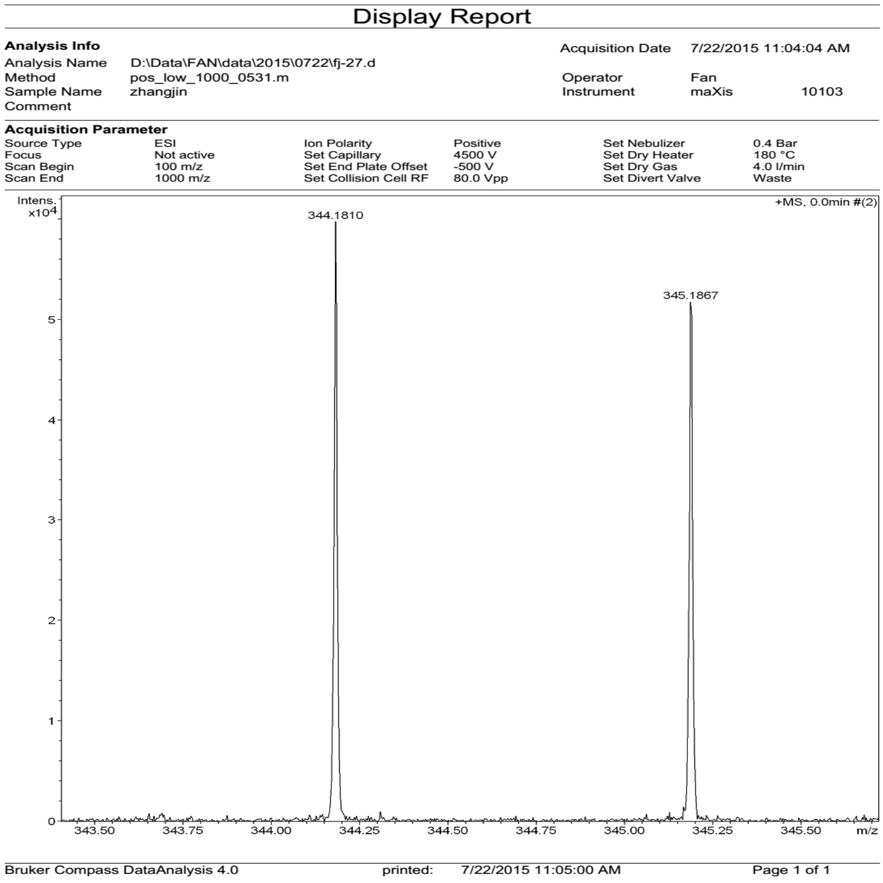

Figure S1. HRESIMS of compound **1**.

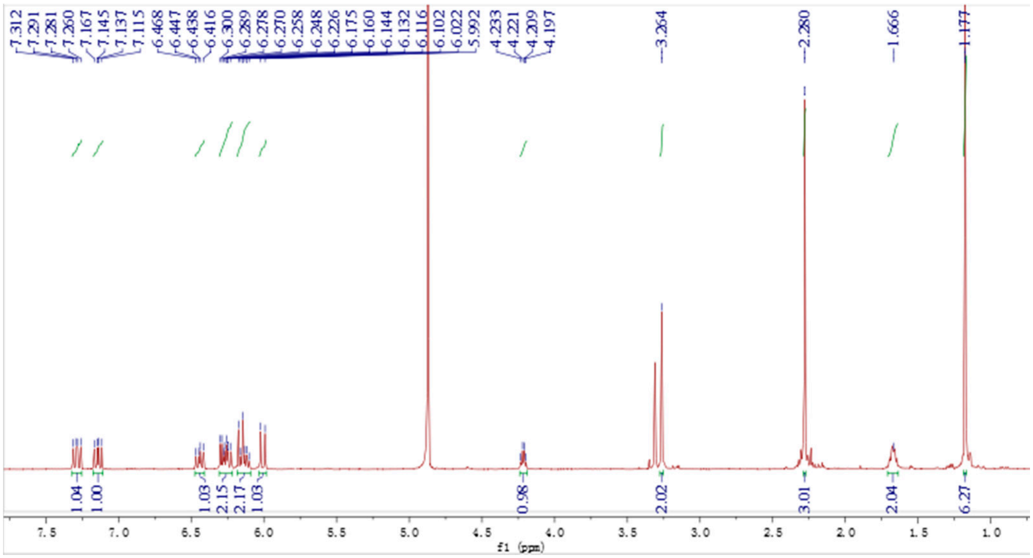

Figure S2. ^{1}H-NMR spectrum of compound **1** in MeOH-*d*$_6$.

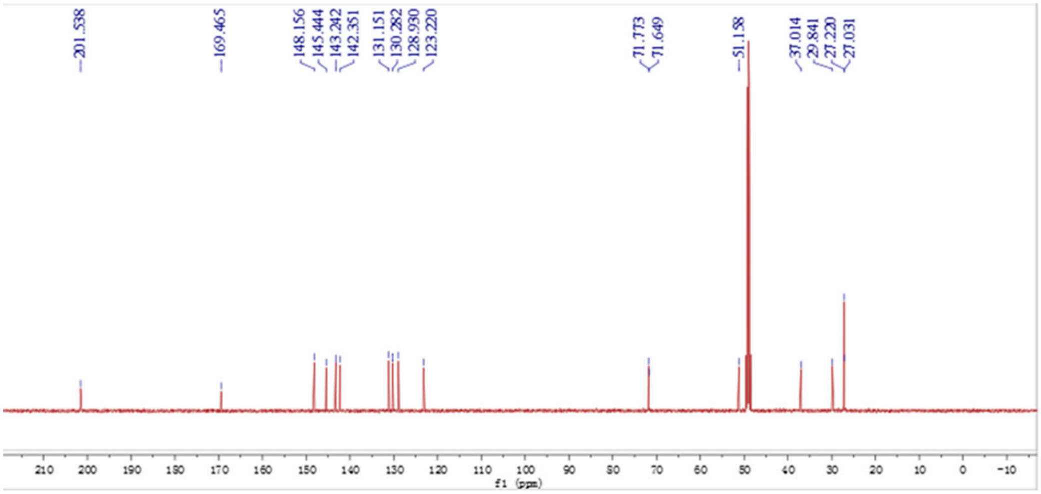

Figure S3. ^{13}C-NMR spectrum of compound **1** in MeOH-d_6.

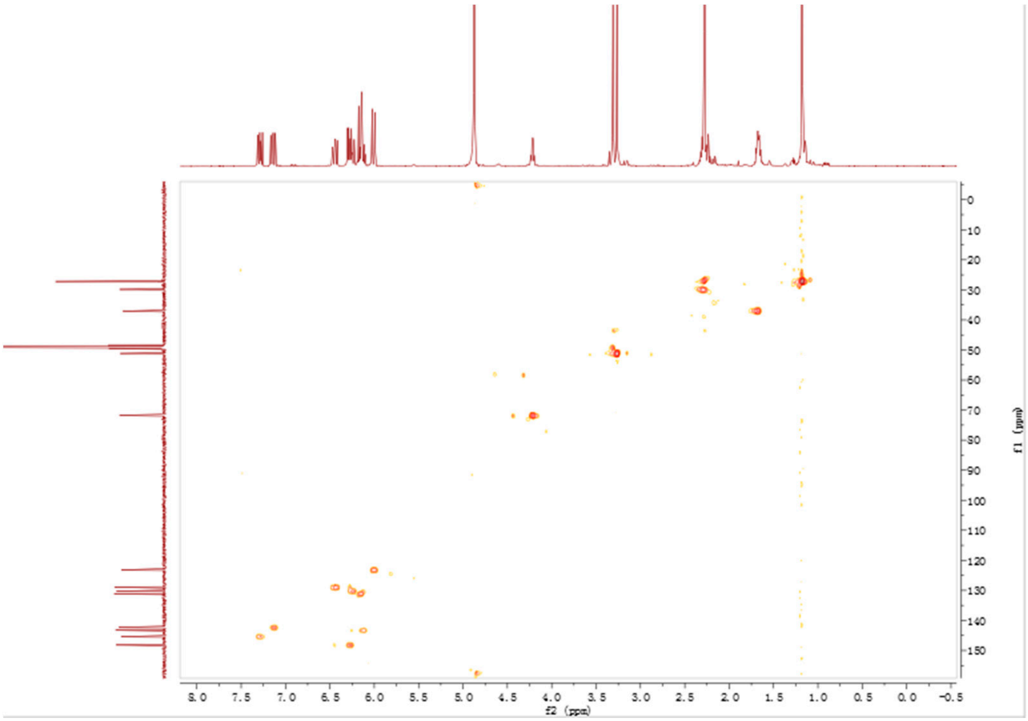

Figure S4. HSQC spectra of compound **1** in MeOH-d_6.

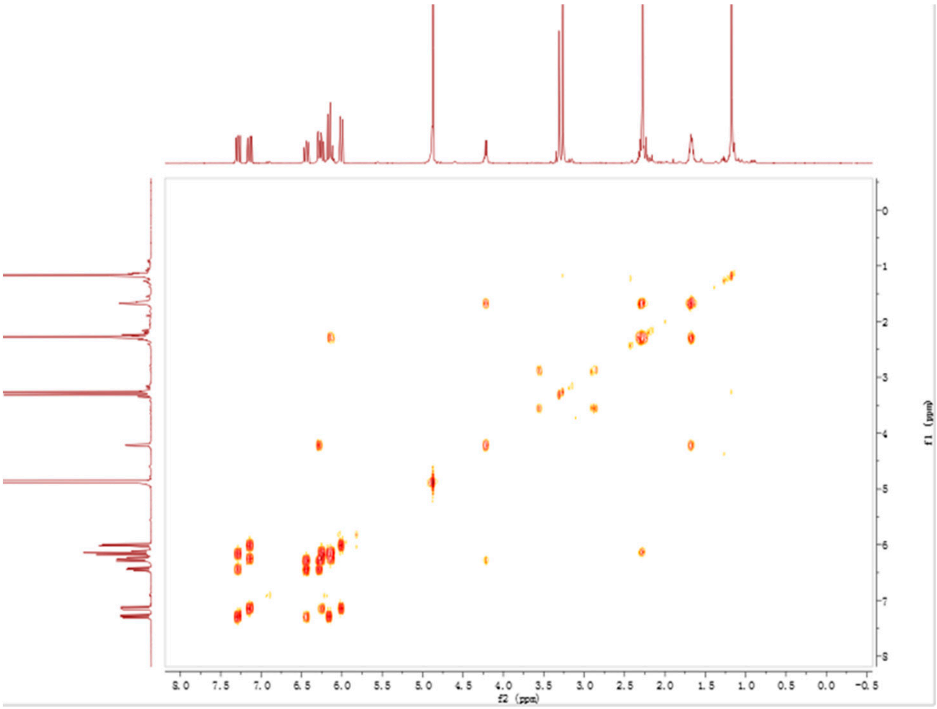

Figure S5. ^{1}H-^{1}H COSY spectrum of compound **1** in MeOH-d_6.

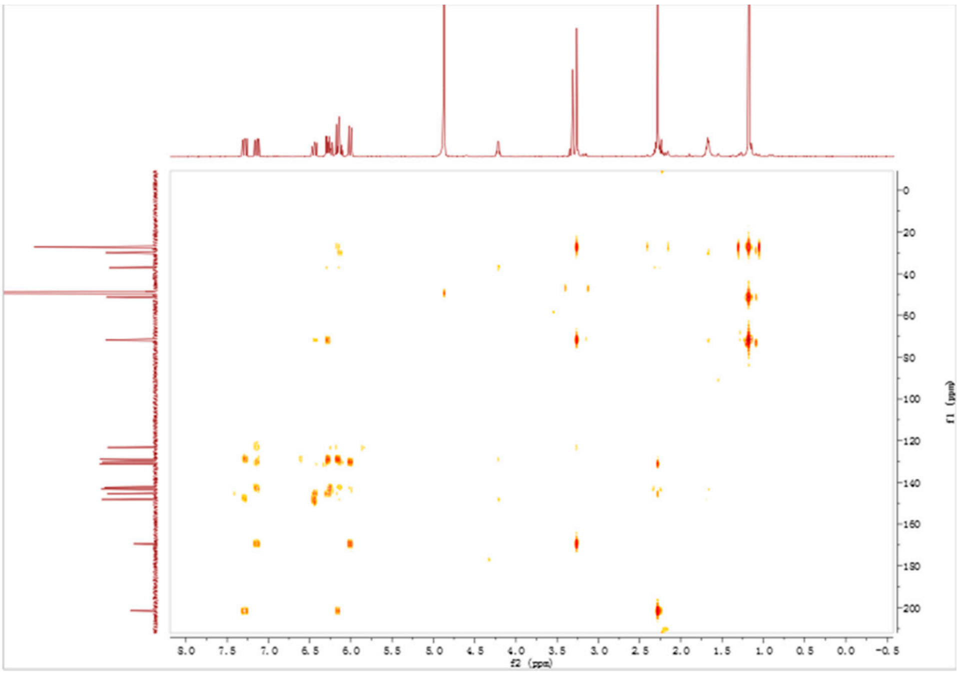

Figure S6. HMBC spectra of compound **1** in MeOH-d_6.

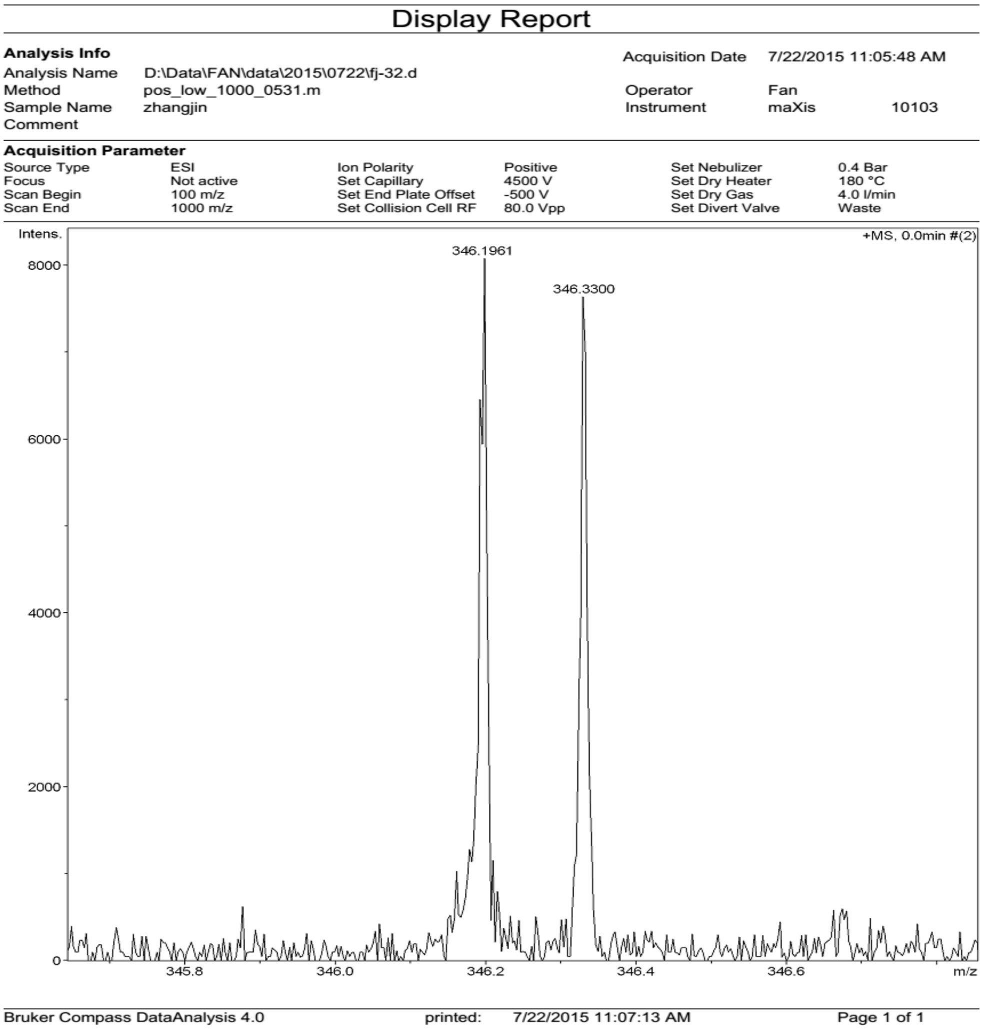

Figure S7. HRESIMS of compound **2**.

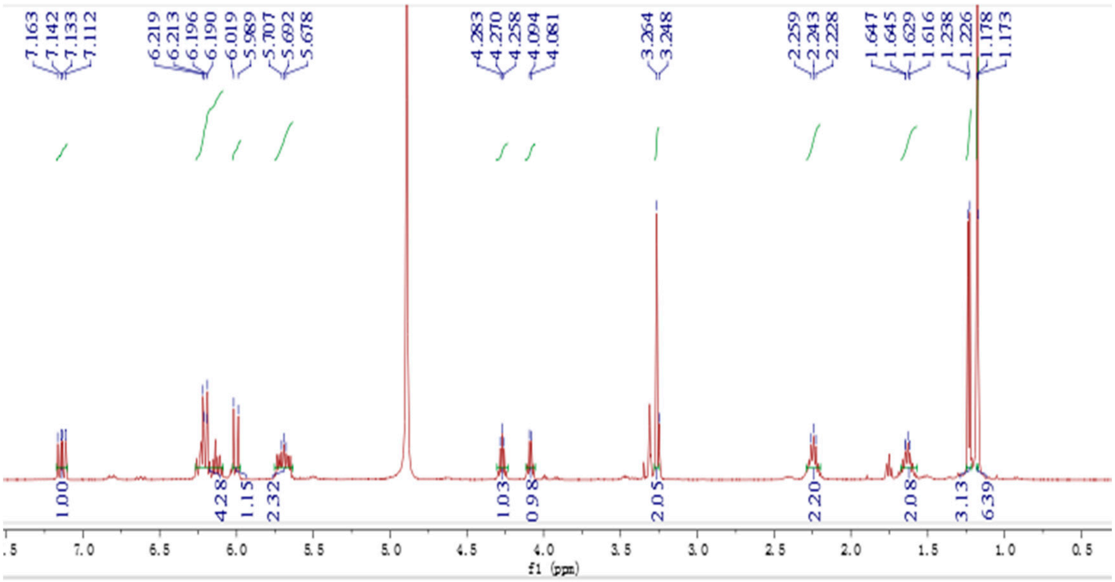

Figure S8. ^{1}H-NMR spectrum of compound **2** in MeOH-d_6.

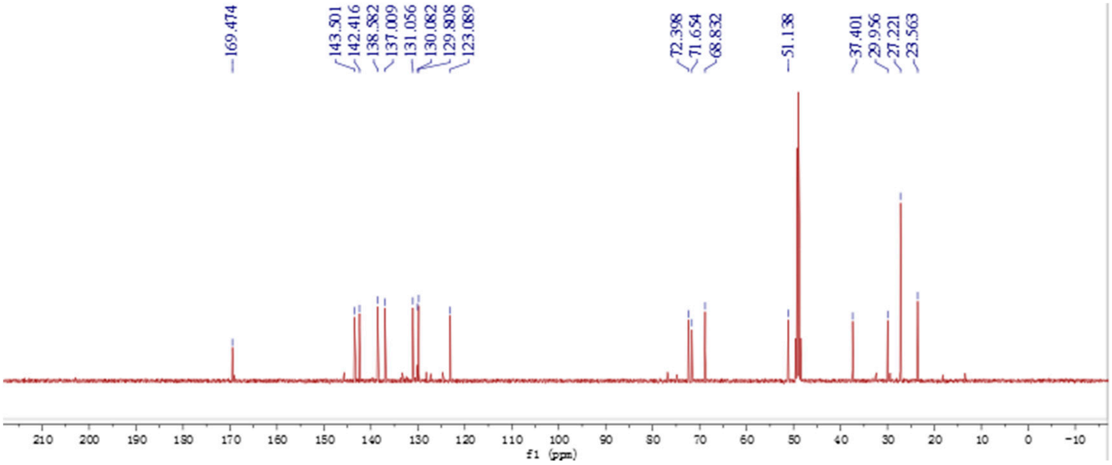

Figure S9. ^{13}C-NMR spectrum of compound **2** inMeOH-d_6.

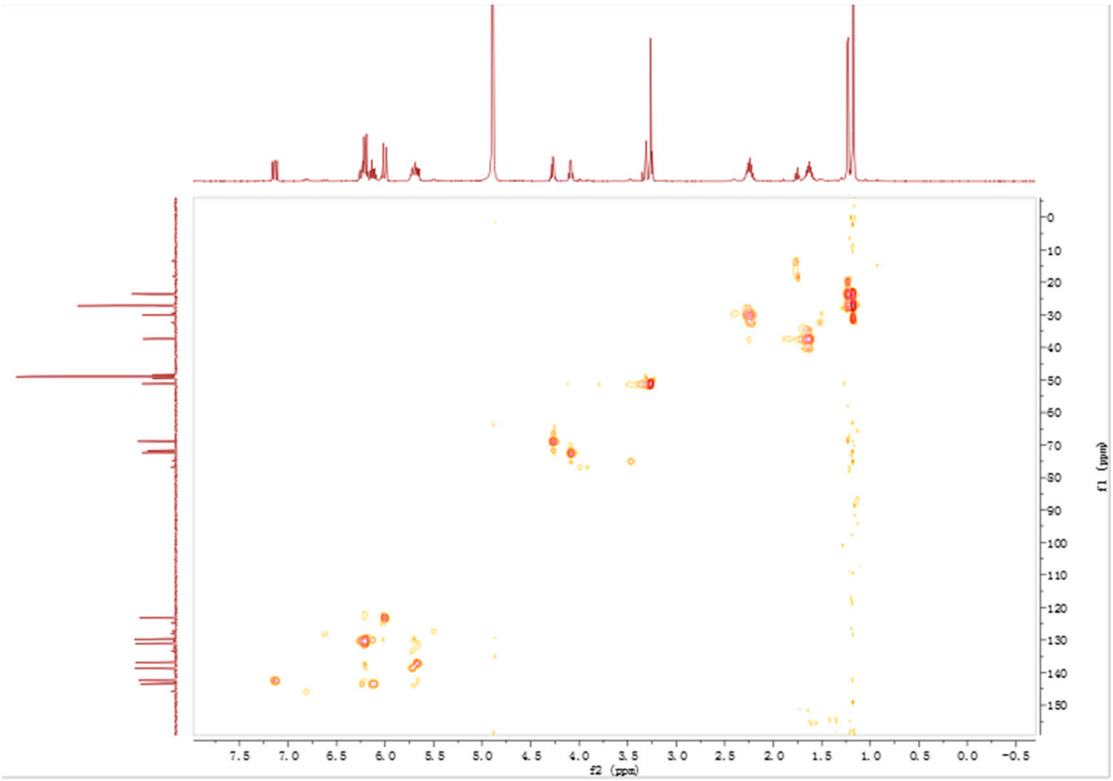

Figure S10. HSQC spectra of compound **2** in MeOH-d_6.

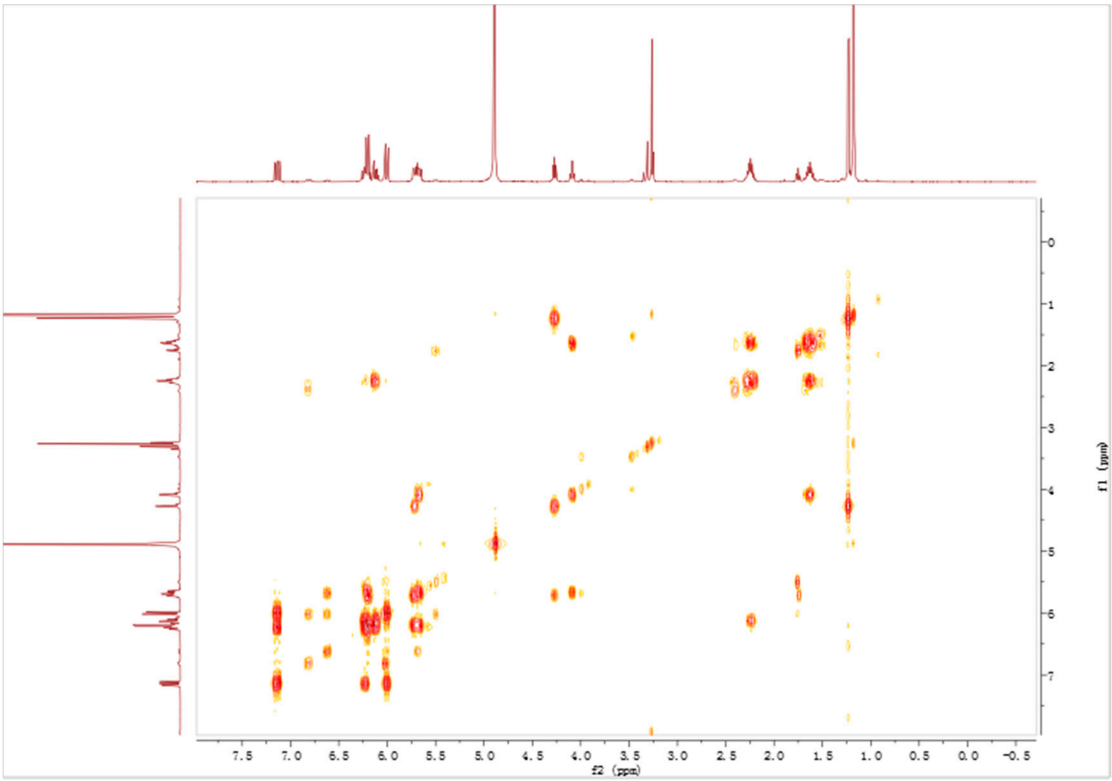

Figure S11. ^1H–^1H COSY spectrum of compound **2** in MeOH-d_6.

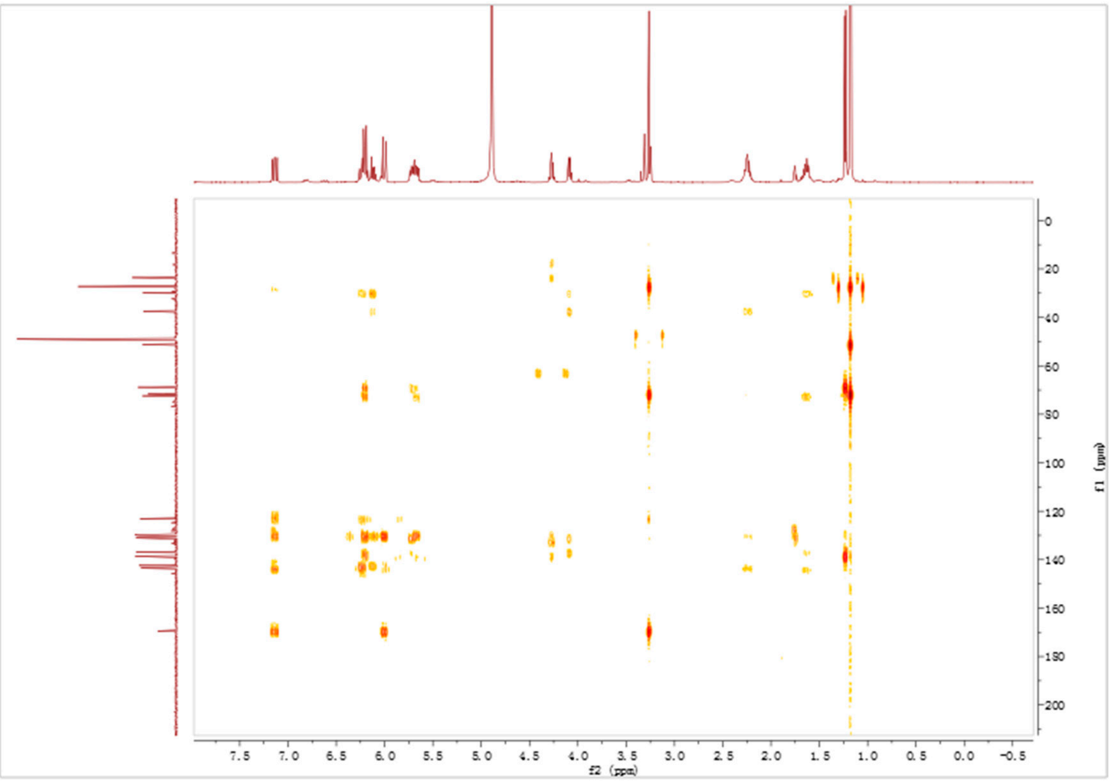

Figure S12. HMBC spectra of compound **2** in MeOH-d_6.

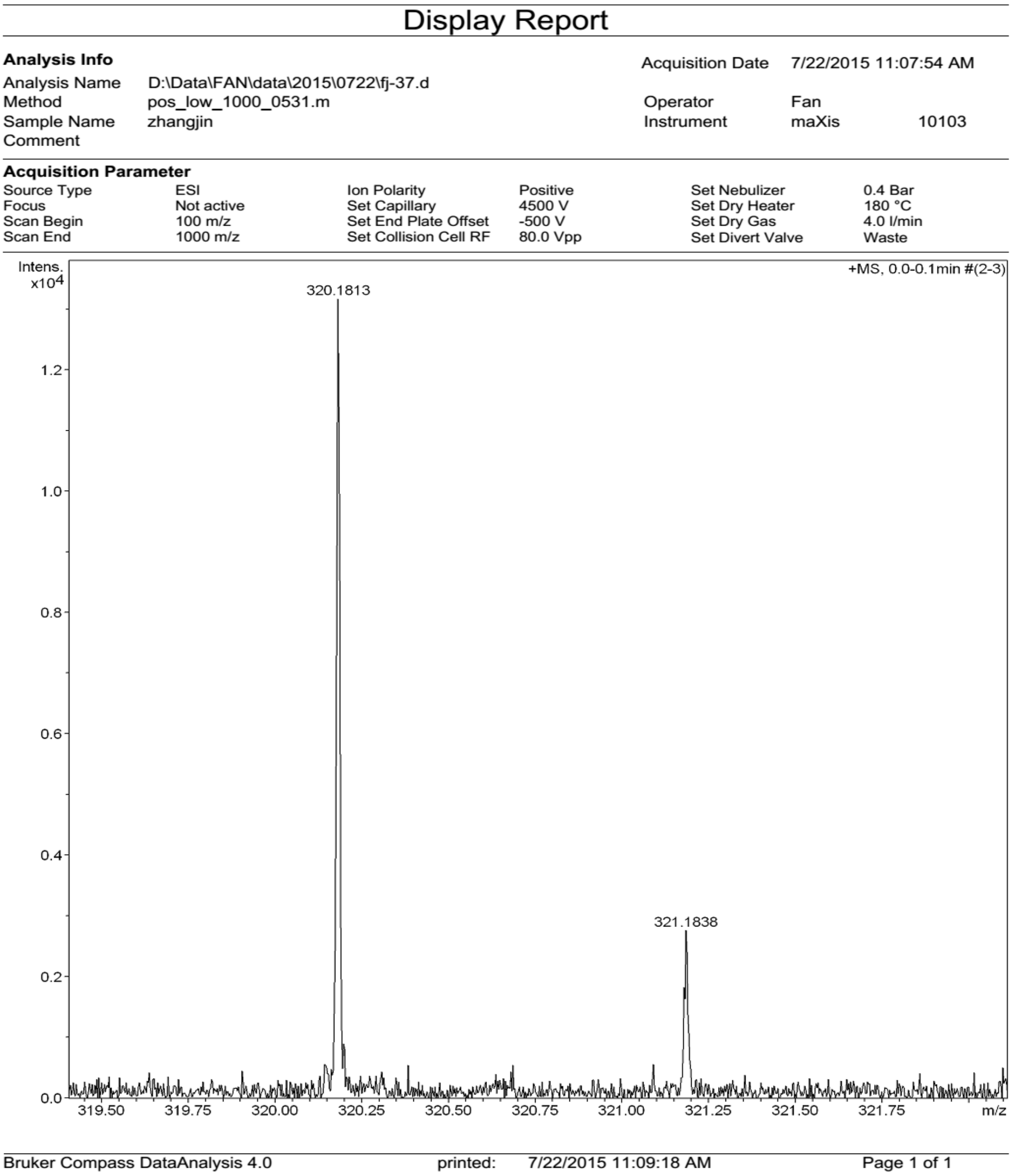

Figure S13. HRESIMS of compound **3**.

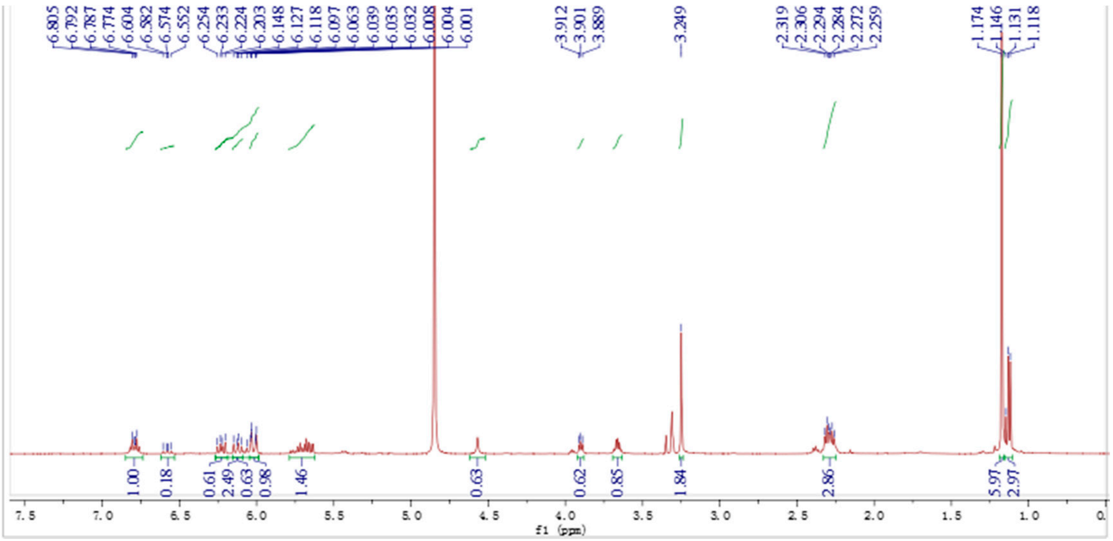

Figure S14. ^{1}H-NMR spectrum of compound **3** in MeOH-d_6.

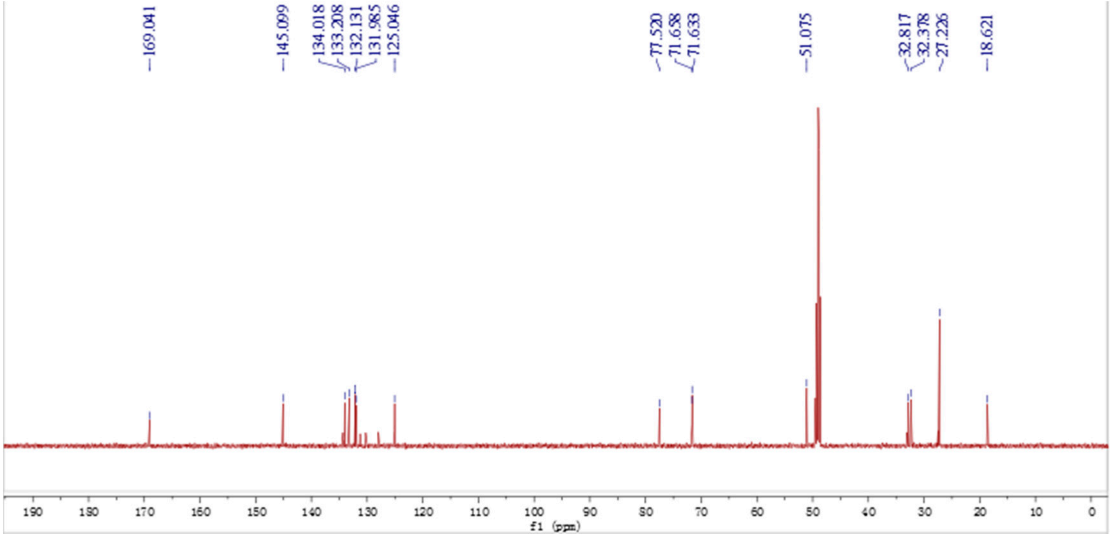

Figure S15. ^{13}C-NMR spectrum of compound **3** in MeOH-d_6.

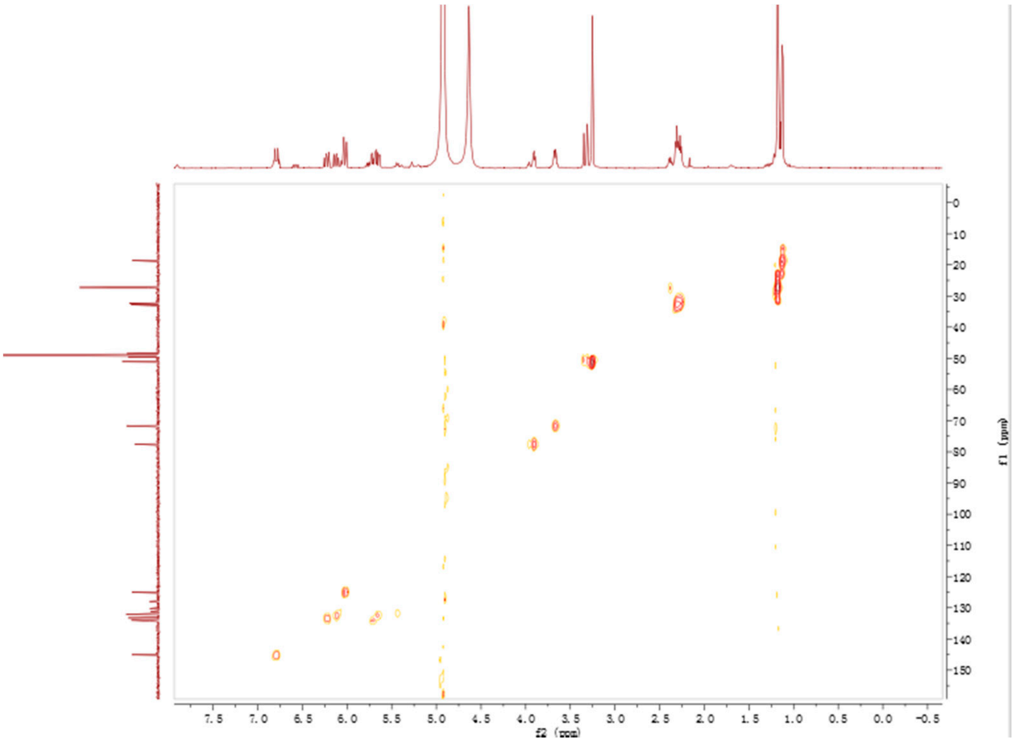

Figure S16. HSQC spectra of compound **3** in MeOH-d_6.

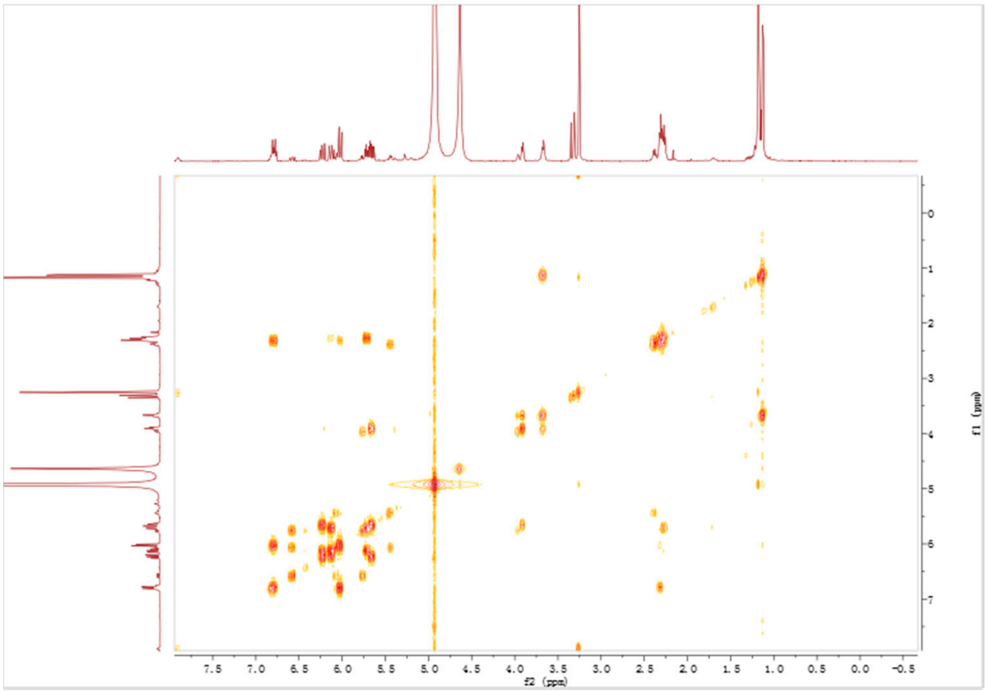

Figure S17. ^{1}H-^{1}H COSY spectrum of compound **3** in MeOH-d_6.

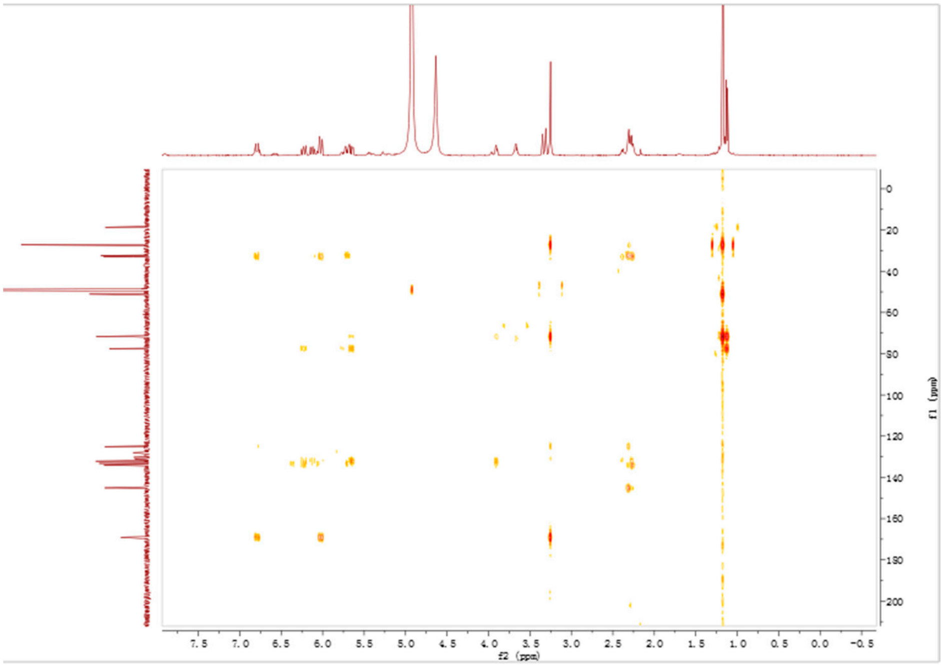

Figure S18. HMBC spectra of compound **3** in MeOH-d_6.

Display Report

Analysis Info

Analysis Name D:\Data\FAN\data\2015\0722\fj-4-1.d
Method pos_low_1000_0531.m
Sample Name zhangjin
Comment

Acquisition Date 7/22/2015 11:12:24 AM
Operator Fan
Instrument maXis 10103

Acquisition Parameter

Source Type	ESI	Ion Polarity	Positive	Set Nebulizer	0.4 Bar
Focus	Not active	Set Capillary	4500 V	Set Dry Heater	180 °C
Scan Begin	100 m/z	Set End Plate Offset	-500 V	Set Dry Gas	4.0 l/min
Scan End	1000 m/z	Set Collision Cell RF	80.0 Vpp	Set Divert Valve	Waste

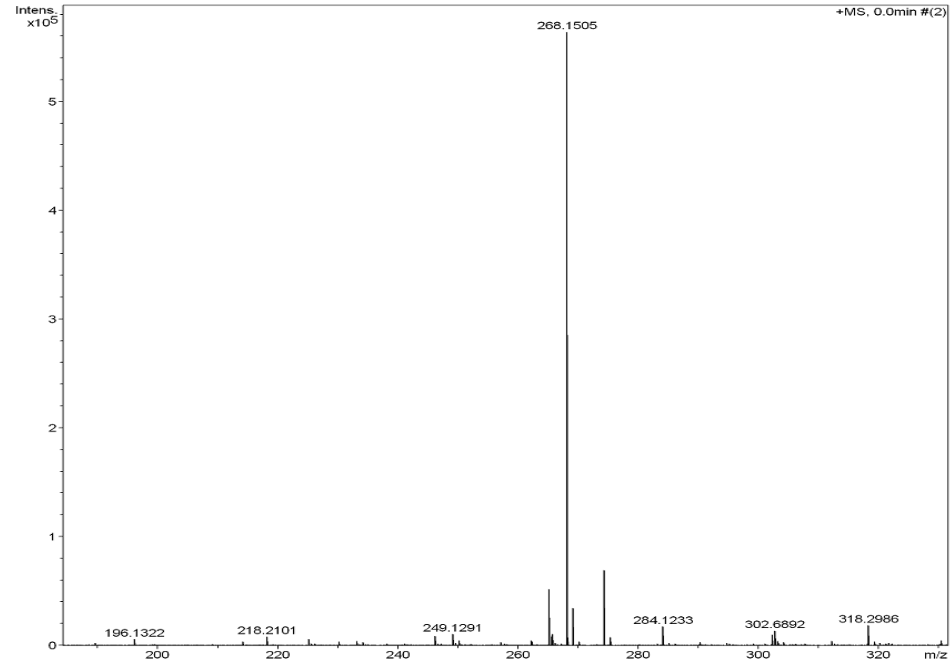

Bruker Compass DataAnalysis 4.0 printed: 7/22/2015 11:13:41 AM Page 1 of 1

Figure S19. HRESIMS of compound **4**.

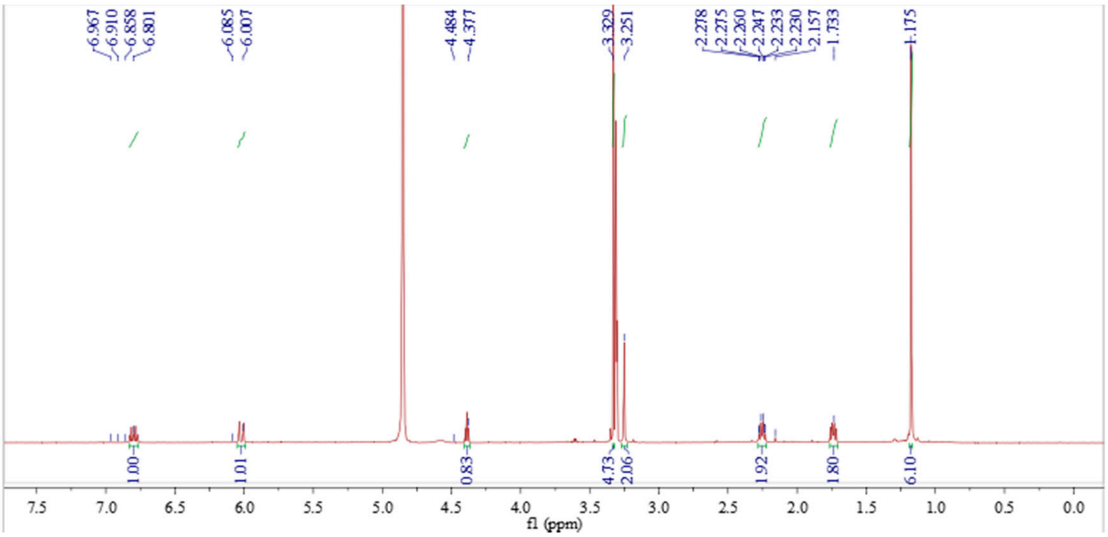

Figure S20. ^{1}H-NMR spectrum of compound **4** in MeOH-d_6.

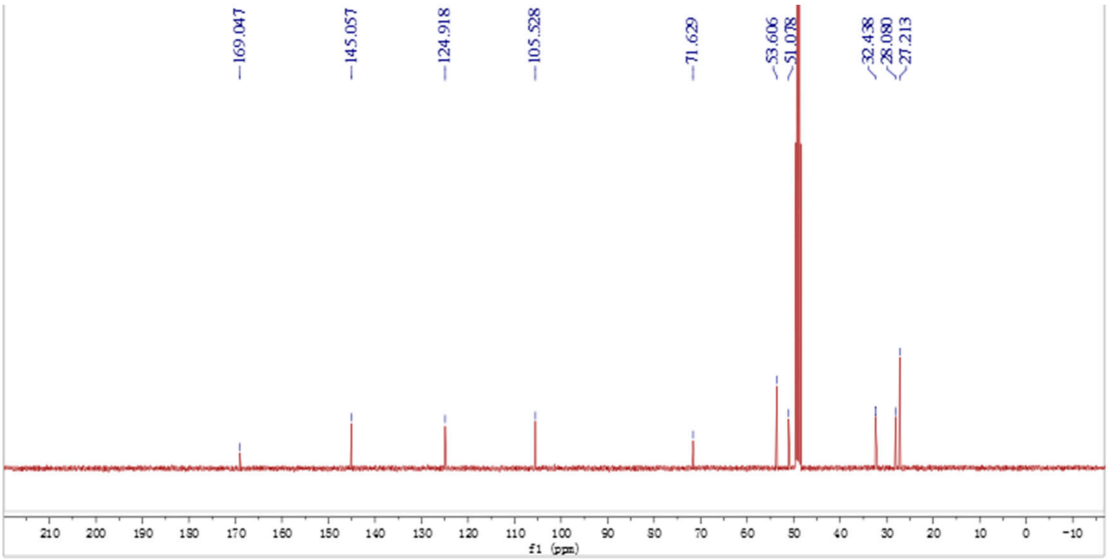

Figure S21. ^{13}C-NMR spectrum of compound **4** in MeOH-d_6.

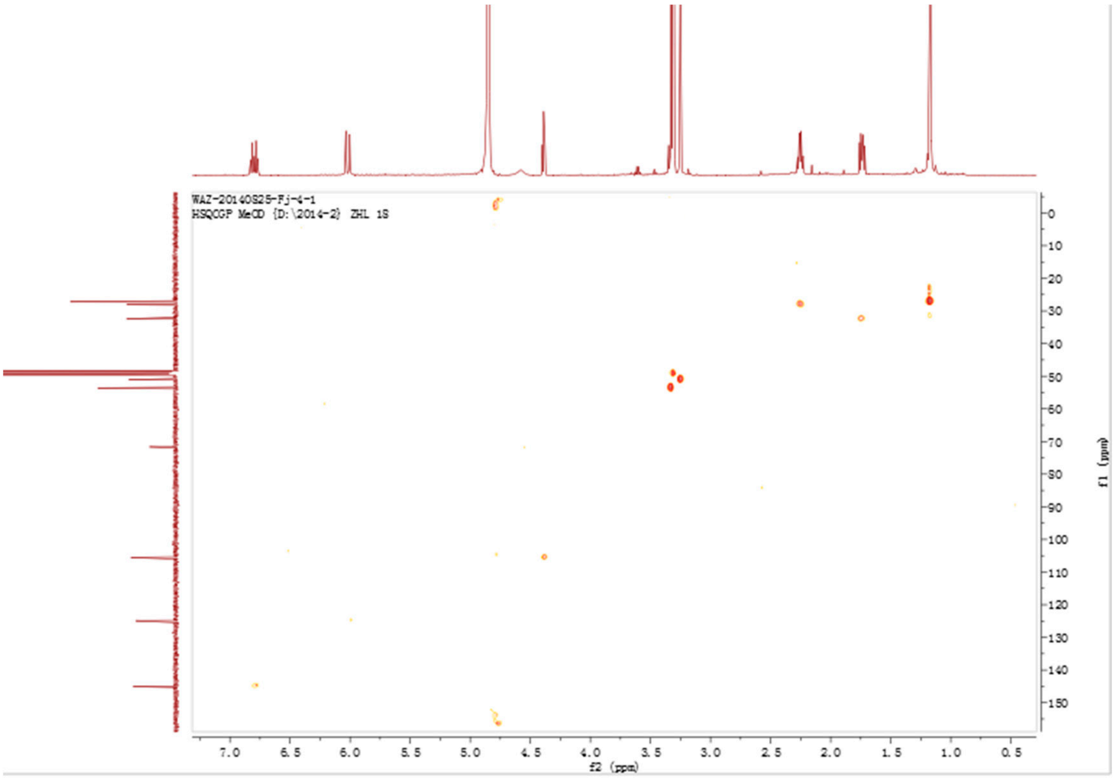

Figure S22. HSQC spectra of compound **4** in MeOH-d_6.

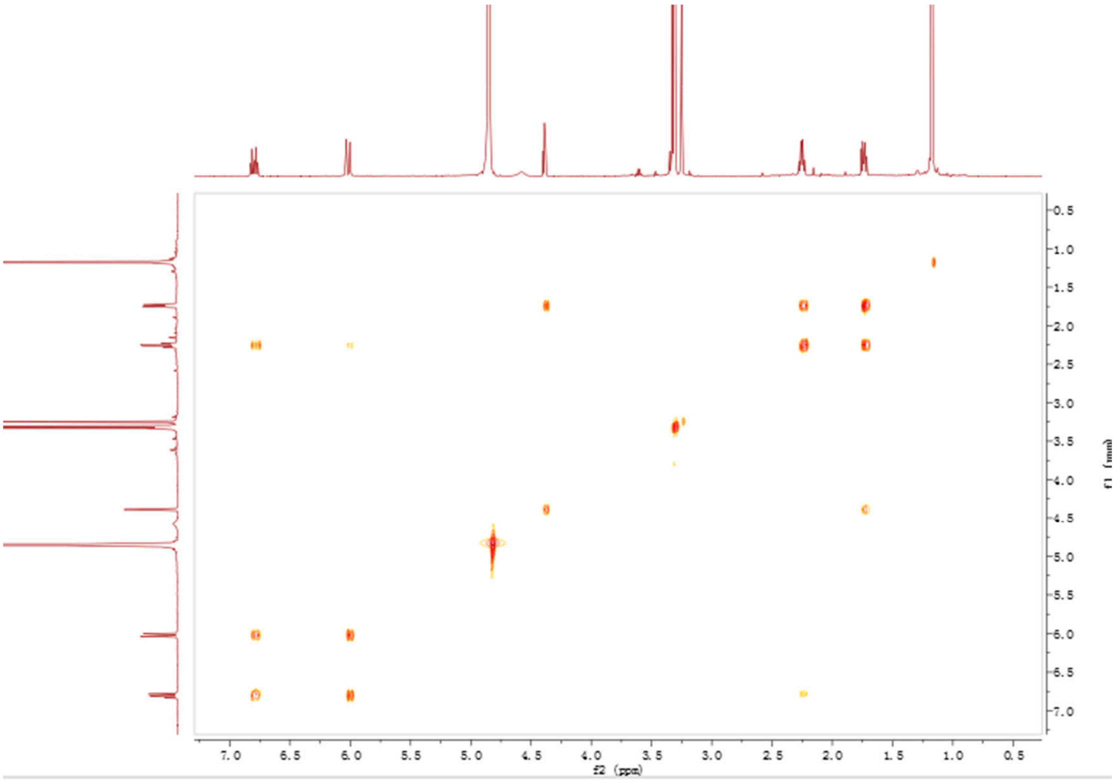

Figure S23. ^{1}H-^{1}H COSY spectrum of compound **4** in MeOH-d_6.

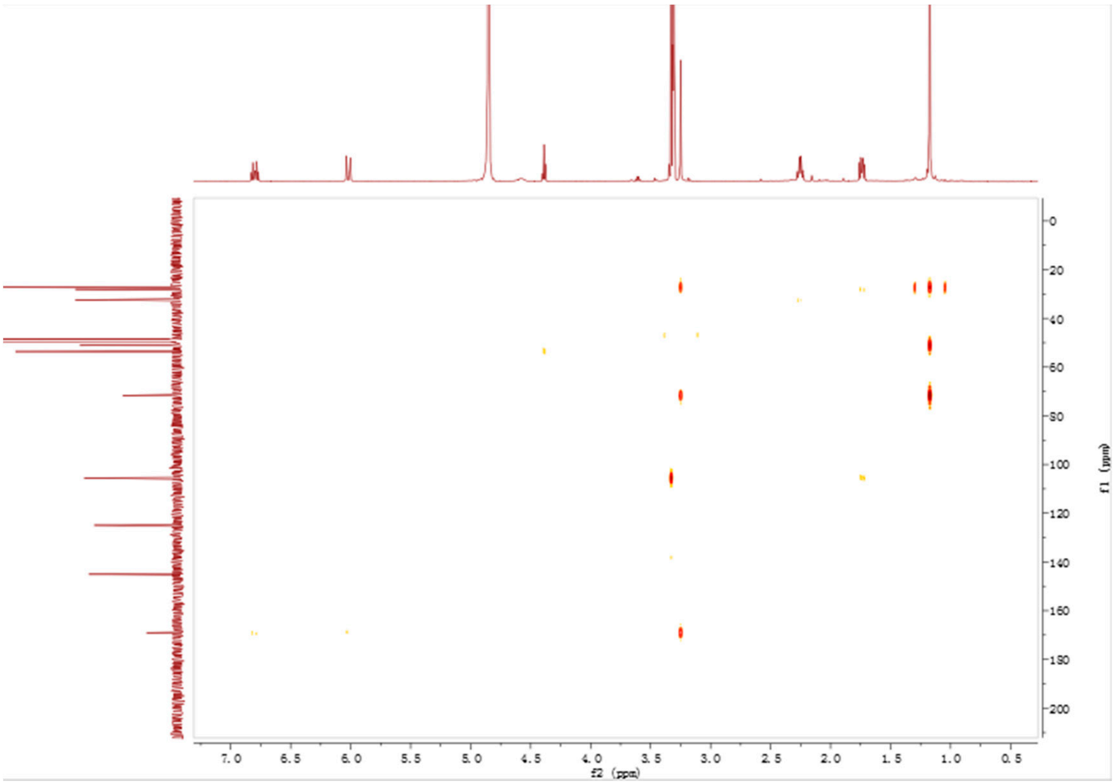

Figure S24. HMBC spectra of compound **4** in MeOH-d_6.

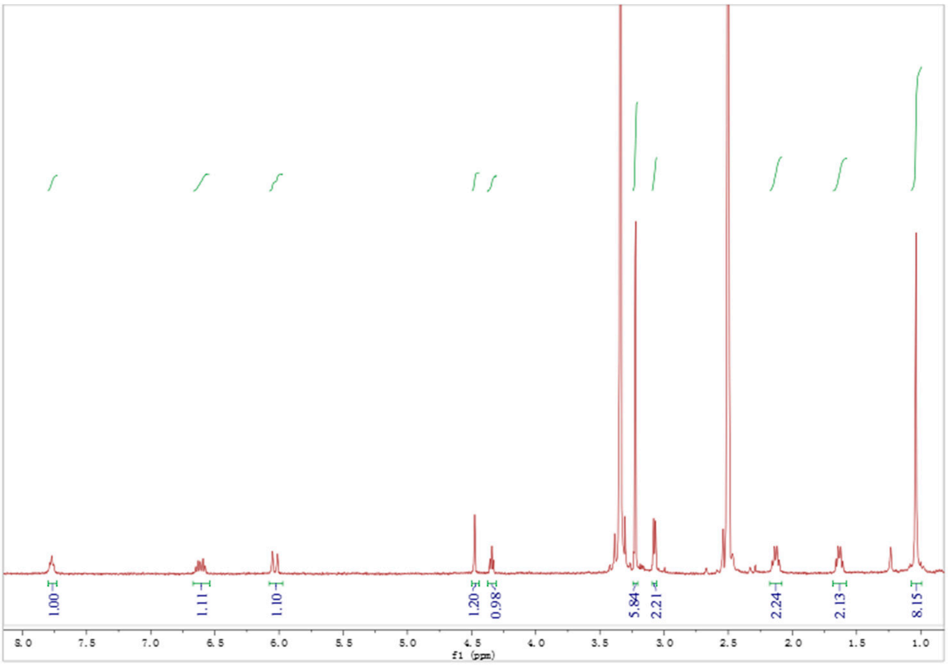

Figure S25. ^{1}H-NMR spectrum of compound **4** in DMSO-d_6.
